# Modulation of Compartmentalised Cyclic Nucleotide Signalling via Local Inhibition of Phosphodiesterase Activity

**DOI:** 10.3390/ijms17101672

**Published:** 2016-10-02

**Authors:** Marcella Brescia, Manuela Zaccolo

**Affiliations:** Department of Physiology, Anatomy and Genetics, University of Oxford, Oxford OX1 3TP, UK; marcella.brescia@dpag.ox.ac.uk

**Keywords:** cAMP, cGMP, Compartmentalisation, Phosphodiesterases (PDEs), PDE inhibitor, protein kinase A, signaling

## Abstract

Cyclic nucleotide phosphodiesterases (PDEs) are the only enzymes that degrade the cyclic nucleotides cAMP and cGMP, and play a key role in modulating the amplitude and duration of the signal delivered by these two key intracellular second messengers. Defects in cyclic nucleotide signalling are known to be involved in several pathologies. As a consequence, PDEs have long been recognized as potential drug targets, and they have been the focus of intense research for the development of therapeutic agents. A number of PDE inhibitors are currently available for the treatment of disease, including obstructive pulmonary disease, erectile dysfunction, and heart failure. However, the performance of these drugs is not always satisfactory, due to a lack of PDE-isoform specificity and their consequent adverse side effects. Recent advances in our understanding of compartmentalised cyclic nucleotide signalling and the role of PDEs in local regulation of cAMP and cGMP signals offers the opportunity for the development of novel strategies for therapeutic intervention that may overcome the current limitation of conventional PDE inhibitors.

## 1. Introduction to Cyclic Nucleotides

Significant progress has been made since 1958, when Rall and Sutherland first described the involvement of adenosine 3′-5′-cyclic monophosphate (cAMP) in the cellular response to hormones [1,2]. Today it is well known that cAMP and guanosine 3′-5′-cyclic monophosphate (cGMP) are intracellular second messengers involved in several cellular processes, including secretion, energy metabolism, gene transcription, cell migration, growth, and survival, and several other cell-specific functions, including immune function, learning and memory, and cardiac strength and frequency of contraction [3,4]. Defects in cyclic nucleotide (CN) signalling are known to be involved in the pathogenesis of multiple diseases, and several drugs currently in use target cAMP or cGMP signalling pathways [5].

cAMP is generated by the activation of adenylyl cyclases (ACs). This activation occurs by the binding of a first messenger (a hormone or neurotransmitter) to G protein-coupled receptors (GPCR), which triggers the release of the Gs α-subunit from the heterotrimeric G protein complex. The Gs α-subunit activates transmembrane ACs (tmACs) for the generation of cAMP by the conversion of ATP [2]. cAMP can also be generated by a soluble adenylyl cyclase (sAC). sAC is distinct from tmACs, as it is regulated by bicarbonate and Ca^2+^ and is present in the cytosol and in several intracellular organelles and structures [6]. The effectors of cAMP are protein kinase A (PKA), which phosphorylates downstream targets at serine and threonine residues; the GTPase Exchange Factor directly activated by cAMP (EPAC), which functions as a nucleotide exchange factor for the Rap subfamily of RAS-like small GTPases; cyclic nucleotide-gated ion channels; and phosphodiesterases (PDEs), the enzymes that degrade CN.

cGMP is generated by two different types of guanylyl cyclase (GC). Membrane-associated (or particulated) guanylyl cyclases are activated by the atrial natriuretic peptide, the B-type natriuretic peptide, and the C-type natriuretic peptide. Soluble GC is activated by nitric oxide, a gaseous signalling molecule endogenously synthesized from l-arginine by nitric oxide synthases. Both particulate and soluble GCs generate cGMP by the conversion of GTP. The downstream effectors of cGMP are protein kinase G (PKG), ion channels, and PDEs.

Non-canonical CN (such as cCMP and cUMP) can also be generated by the sGC and sAC, and share downstream effectors (PKA and PKG) with the classical CN, cAMP, and cGMP [7]. cCMP and cUMP have recently emerged as intracellular second messengers [7]. Relatively little information is available on their physiological functions. However, studies have suggested their involvement in several biological processes, such as cell death when cUMP is generated in combination with cGMP [8], vasodilation, and inhibition of platelet aggregation via PKG phosphorylation in response to cCMP [9]. A detailed account of the regulation of non-canonical CN is outside our scope, but excellent reviews on these emerging intracellular messengers are available elsewhere [7,10].

PDEs are the only enzymes that degrade CN, and are grouped in a superfamily comprising 21 genes, which constitute 11 distinct PDE families, PDE1–11. Each PDE family may include several genes, and at least 100 different PDE isoforms can be generated by different splice variants or alternative transcription start sites [11]. Each isoform shows unique substrate specificity and affinity, kinetic properties, regulatory mechanisms, and subcellular localisation [12]. PDE1–3, 10, and 11 hydrolyse both cAMP and cGMP, although each of their isoforms presents different affinities for CN. PDE1A, PDE1B, and PDE10A have a higher affinity for cGMP over cAMP, whereas PDE1C, PDE2A, and PDE11A have equal affinity for both CNs. PDE3A and PDE3B present similar affinity for cAMP and cGMP, but much higher catalytic rates for cAMP than for cGMP. PDE4, 7, and 8 degrade only cAMP, and PDE5, 6, and 9 are cGMP-specific [13,14]. PDEs are subject to a complex system of regulatory mechanisms. The activity of some PDEs is regulated by post-translational modification. For example, the N-terminal domain of PDE1–5 can undergo phosphorylation, and PDE4 isoforms can be ubiquitinated at their C-terminus [15]. PDE1 is regulated by intracellular Ca^2+^. This enzyme includes a Ca^2+^/calmodulin binding site at the N-terminus, and binding of Ca^2+^/CaM leads to 10-fold stimulation of its maximal velocity (V_max_) without changing its affinity for the substrate. However, the Michaelis constant K_m_ for the substrate is reduced when the same site is phosphorylated by PKA [16]. PDE2, 6, and 9 contain allosteric binding sites for cGMP (called GAF domains) from cGMP-binding PDEs *Anabaena* adenylyl cyclase and *Escherichia coli* Fh1A, which function as ligand-binding domains or facilitators of protein–protein interaction [17,18]. Binding of cGMP to PDE2 and PDE5 GAF domains increases the hydrolytic activity of the enzyme. This is particularly interesting, as it allows cross-talk between the cAMP and cGMP pathways with possible reciprocal regulation. cGMP activates PDE2, which, as previously discussed, degrades both cAMP and cGMP. Therefore, the intracellular levels of cGMP can influence the rate at which PDE2 hydrolyses cAMP. cGMP can also stimulate PDE5 by binding to its GAF domain, and thus it can increase the rate of its own degradation. In addition, cGMP binding to PDE5 promotes PKG-mediated phosphorylation, which again increases PDE5 enzymatic activity. This PDE5 regulatory mechanism does not seem to be cGMP-specific, as PKA-mediated phosphorylation appears to have a similar effect [17,19,20]. The regulation of PDE3 is also involved in the interconnection between cAMP and cGMP signalling. This enzyme has dual-specificity and binds with high affinity both cAMP and cGMP, which are mutually competitive substrates. Because PDE3 shows a much higher catalytic rate for cAMP than for cGMP, PDE3 functions principally as a cGMP-inhibited cAMP-hydrolysing enzyme. Consequently, the levels of cGMP can alter the availability of PDE3 to degrade cAMP, thus regulating cAMP concentration. PDE3 can be phosphorylated by PKA, and this phosphorylation enhances its activity [17,21]. The complex control system illustrated above differentially regulates the activity of the multiplicity of PDE isoforms and provides a means to fine-tuning CN levels in response to the continuously changing requirements of the cell [22,23].

## 2. Compartmentalisation of Cyclic Nucleotides

The model initially proposed for cAMP signalling was simple and linear: the first messenger activates a GPCR, and cAMP is generated, leading to the activation of PKA. The PKA-mediated phosphorylation of downstream protein targets then results in the required cellular effect [24]. However, the idea that cAMP could activate PKA, which in turn could phosphorylate a multiplicity of proteins without any selectivity appeared to be unsatisfactory since the early days [4]. As further research uncovered the complexity of the cAMP signalling pathway, it became apparent that a more sophisticated model was required. The challenge was to reconcile the fact that the same cell can express multiple GPCRs, all signalling via cAMP, and that PKA can phosphorylate a vast number of protein targets within the same cell with the ability of the cell to effectively coordinate its response to a specific extracellular stimulus and achieve the required functional outcome with high fidelity [4]. To resolve this conundrum, in the early 1980s, the concept was put forward that cAMP signalling must be compartmentalised. Brunton and co-workers observed that the stimulation of cardiac myocytes with either prostaglandin E1 (PGE1) or isoproterenol resulted in the generation of cAMP, but yielded very different functional outcomes: isoproterenol caused an enhanced force of contraction, whereas this effect was not detected when the heart was perfused with PGE1 [25]. To explain this observation, it was suggested that distinct subsets of PKA are activated in response to different stimuli, thus allowing for hormonal specificity of cAMP signalling [26]. However, a mechanistic understanding of how this could happen remained elusive for several decades.

**Figure 1:**
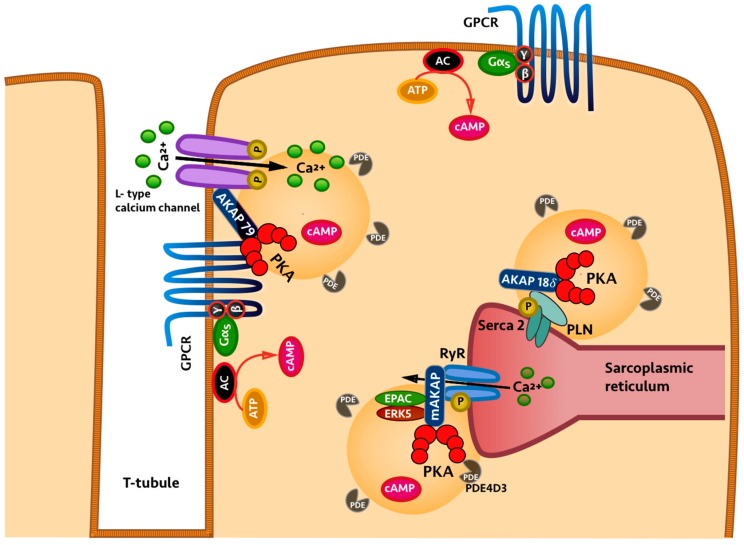
Compartmentalised adenosine 3′-5′-cyclic monophosphate (cAMP) signalling by AKAPs (A kinase anchoring proteins) and phosphodiesterases (PDEs) and intracellular distribution of three AKAP complexes involved in cardiac contractility. AC: adenylyl cyclase; EPAC: GTPase exchange factor directly activated by cAMP; GPCR: G protein-coupled receptor; P: phosphate group; PKA: protein kinase A; PLN: Phospholamban; RyR: Ryanodine receptor. Red shaded areas indicate distinct cAMP pools generated by an AC anchored at the plasma membrane and activated by a GPCR exposed to the extracellular space. Black arrows indicate Ca^2+^ flow. Red arrows indicate synthesis of cAMP. PKA anchored to AKAPs and the activity of PDEs contribute to spatial regulation of cAMP and specific downstream physiological effect, as selective PKA phosphorylates the target that is contained within the specific microdomain.

Research over the past 30 years has clearly established that CN signalling is indeed compartmentalised [22]. Compartmentalised signalling results from the ability of individual GPCRs to generate spatially-distinct pools of cAMP. These in turn activate defined subsets of localised PKA, which are tethered in proximity to specific targets via binding to anchoring proteins. PDEs play a key role in the spatial regulation of cAMP propagation. They not only contribute to the establishment of boundaries to cAMP diffusion and to the generation of cAMP pools where the second messenger is confined within delimited subcellular compartments, but they also regulate cAMP levels within individual compartments [22]. A-kinase anchoring proteins (AKAPs) are scaffolding proteins that anchor PKA to specific subcellular sites and are instrumental in keeping cAMP signalling specific and physically compartmentalised. AKAPs form signalling hubs (or signalosomes) which organise within the same macromolecular complex GPCR, AC, PDEs, PKA and its targets, and phosphatases, ensuring selective phosphorylation and tight local regulation of signal duration [27]. More than 50 AKAPs and their strategic localisation have already been identified. For example, in the heart, several AKAPs involved in the regulation of excitation–contraction coupling have been described. The localisation of AKAP79 at the plasmalemma is required for PKA-mediated phosphorylation of l-type calcium channels (LTCC) in response to β-adrenergic receptor stimulation [28]. LTCCs are essential in the generation of normal cardiac rhythm and in triggering atrial and ventricular contraction, and AKAP79—by tethering PKA in proximity to the channel—plays a crucial role in coordinating the adrenergic regulation of cardiac function [29]. As another example, AKAP18δ interacts with the sarcoplasmic/endoplasmic reticulum calcium ATPase 2 (SERCA2) at the sarcoplasmic reticulum (SR) and ensures the PKA-dependent phosphorylation of SERCA2-associated phospholamban. The disruption of this complex by AKAP18δ knock down compromises the potentiation of Ca^2+^ re-uptake into the SR induced by adrenergic stimulation, confirming that the AKAP18δ/PKA/SERCA2/phospholamban signalosome provides a mechanism for the precise spatiotemporal control of phospholamban phosphorylation [30]. Muscle specific A kinase anchoring protein (mAKAP) is localised in close proximity to the ryanodine receptor (RyR) and also serves as a scaffold to anchor PDE4D3, EPAC1, and the extracellular signal-regulated kinase 5 (ERK5) in cardiac myocytes (Figure 1) [31]. Phosphorylation of RyRs, mediated by mAKAP-anchored PKA, has been implicated in the regulation of cardiac myocyte contractility [28,32,33].

cGMP signalling presents mechanisms of spatial organisation similar to cAMP, yet less is known about the details of its compartmentalisation. A role for PDEs in local cGMP regulation has been demonstrated, in particular for PDE5 and PDE2. There is evidence that these enzymes are differently engaged in the degradation of cGMP, depending on whether cGMP is generated via particulate or soluble GCs, thus suggesting spatial segregation of these cGMP signalling components; PDE9A, unlike PDE5A, regulates cGMP produced via natriuretic peptide rather than nitric oxide stimulation [23,34]. PKG and GCs are suggested to be organized in macromolecular complexes, and the localisation of PDEs appears to be important in determining cGMP microdomains [35]. Although the presence of anchoring proteins for PKG has not yet been firmly established, studies have suggested that proteins like myosin, atrial natriuretic peptide receptor, and troponin T could act like GKAPs [36].

## 3. Role of Phosphodiesterases in Cyclic Nucleotide Compartmentalisation

The key role of PDEs in the compartmentalisation of CN signalling was firmly established using fluorescence resonance energy transfer (FRET)-based genetically encoded fluorescent indicators that allow for cAMP [37,38,39] or cGMP [40,41,42,43] dynamics to be visualized in real-time in intact living cells. By using this technique, it was possible to directly demonstrate for the first time that cAMP generated by β-adrenergic stimulation does not homogeneously equilibrate in the cell, but rather is compartmentalised within defined subcellular microdomains. Upon the inhibition of PDEs, the localisation of cAMP was disrupted, and the second messenger was able to uniformly distribute in the cytosol of cardiac myocytes. The same effect was observed when the cells were pretreated with the inhibitor followed by β-adrenergic stimulation, leading to the conclusion that PDEs control cAMP diffusion and concentration in the cytosol, consequently regulating the local activation of PKA [44]. A body of work supports the involvement of PDEs in the compartmentalisation of cAMP signalling. The cAMP-specific PDE4 family was the focus of some of these studies. PDE4D3 was shown to be compartmentalised with PKA via mAKAP anchoring [31], and PKA was shown to be activated only if the cAMP concentration is sufficiently high to overcome the local cAMP-degrading activity exerted by the local PDE [31]. PDE4D5 was found in complex with the scaffold protein β-arrestin, which is recruited to the β2 adrenergic receptor (β2-AR) upon agonist stimulation, where it plays an important role in attenuating the activity of a local pool of PKA tethered to the β2-AR by AKAP79. This local pool of PKA phosphorylates β2-AR, allowing it to switch its coupling from Gs to Gi, leading to the activation of ERK. Therefore, PDE4 in complex with β-arrestin was found to be instrumental in controlling the phosphorylation of β2-AR by AKAP79-tethered PKA [45].

As PDEs are the only route to CN degradation, the specific subcellular localisation of these enzymes represents a fundamental mechanism for the local regulation of the magnitude, duration, and specificity of CN-dependent signals. Without PDE-dependent control of local cAMP levels, stimulation of adenylyl cyclase activity by any hormone would fill up the cells with a uniform concentration of the second messenger. As a consequence, signal specificity would be lost, as all PKA subsets present in the cell would be activated [22]. In this scenario, manipulation of cAMP levels by specific pharmacological inhibition of individual PDE families offers the opportunity to achieve a more specific effect compared to activation of the upstream GPCR, which may trigger a complex downstream signalling cascade.

Pharmacological inhibition of PDEs is extensively used for experimental work, and it has enormously contributed to our understanding of the role of individual PDE families and the definition of the cAMP compartmentalisation model. For example, the PDE2 inhibitor Bay 60-7550 was used to show that PDE2 is responsible for the degradation of cGMP in hippocampal neurons and can improve memory functions by enhancing neuronal plasticity [46]. The same inhibitor was used to demonstrate that a pool of cAMP—selectively modulated by PDE2A—regulates hypertrophic growth of cardiac myocytes [47]. Studies where PDE3 was inhibited with cilostamide and PDE4 with rolipram showed that reducing the total PDE4 activity by only 10% results in a dramatic increase in cAMP, whereas total PDE3 inhibition has only a marginal effect on the cAMP response to β-AR stimulation, suggesting that compartmentalisation of PDEs is as important as their level of expression in determining their impact on CN levels and cell function [48].

A number of PDE inhibitors are used in the clinic. PDE3–5 family-selective inhibitors have been approved for clinical use. For example, roflumilast (a PDE4 selective inhibitor) is indicated for the treatment of chronic obstructive pulmonary disease (COPD). However, PDE4 inhibitors present adverse emetic effects due to the inhibition of PDE4 in the brain and the gut, limiting their use as therapeutics [18,49,50]. The PDE3 inhibitor cilastazol has been widely used to treat intermittent claudication, a peripheral vascular disease causing pain and cramps in the legs [18]. Milrinone (also a PDE3 inhibitor) is effective in increasing cardiac contractility in failing hearts, but it increases mortality in the long term (most commonly as a consequence of arrhythmias and cardiac arrest [16]), limiting its indication to the treatment of patients with refractory acute decompensated heart failure [18]. The most famous PDE inhibitors available on the market are the PDE5 inhibitors sildenafil (Viagra:Pfizer), vardenafil (Levitra:Bayer/Glaxo SmithKline) and tadalafil (Cialis:Lilly) for the treatment of erectile dysfunction [51]. Sildenafil and vardenafil also inhibit PDE6, and for this reason, their use might be accompanied by brief visual disturbances. Other side-effects, such as headache, flushing, and nasal congestions are mild [18]. PDE5 inhibitors have also been approved for the treatment of pulmonary hypertension and have been evaluated for other indications, such as benign prostatic hyperplasia and cardiomyopathies [13,52,53].

## 4. Local Inhibition of Phosphodiesterase Activity

As discussed above, family-selective PDE inhibitors present limitations for clinical use, mainly due to lack of isoform selectivity, resulting in undesirable side effects. Studies are currently underway to circumvent this problem, and we briefly outline some of the existing isoform-selective inhibition strategies below.

After the publication of the PDE4 crystal structure [54], new insights into the PDE4 regulatory domains allowed the visualization of a previously unknown binding mode for PDE4 inhibitors involving the upstream conserved region 2 (UCR2), which is part of the regulatory domain, and the third C-terminal (CR3) control region, present in all four PDE4 families. The same study described the generation of PDE4D allosteric modulators, small molecules interacting with UCR2 and CR3 that partially inhibit the enzymatic activity and cAMP hydrolysis. These allosteric modulators showed positive results when assessed for pro-cognitive, anti-inflammatory, and antidepressant outcomes with reduced side effects when compared to classical PDE4 inhibitors [18,54,55]. After further elucidation of the regulatory mechanisms, a new PDE4 subfamily-selective inhibitor was developed. This compound (defined as an atypical inhibitor) recognizes specific folded conformations within the UCR2 domain and the catalytic domain of the PDE4, rather than interacting exclusively with the catalytic domain. This approach provides the possibility of isoform selectivity by exploiting a Thyr/Phe polymorphism in the UCR2 helix of PDE4 isoforms [56].

Small interfering RNA (siRNA) of individual PDEs is significantly more specific than most available pharmacological inhibitors [57], allowing for the ablation of sub-families of PDEs. Houslay et al. [58] used this technique to selectively deplete PDE4Bs and PDE4Ds in HEK293B2 cells in order to compare the functional outcome of the interaction of β2-AR with β-arrestin. The transfection of the designed oligonucleotides was highly efficient, with ~95% of each of these PDE sub-families selectively knocked down [58]. The same approach was used to deplete PDE7 and PDE8 and examine the influence of gene silencing on gene expression in human osteoblasts [59] and to establish the anti-hypertrophic effect of PDE2 inhibition [47]. Although very effective for research purposes, siRNA is not likely to be easily adapted for manipulation of PDE activity in the clinical setting.

In a scenario where the compartmentalisation of CN signalling is crucial for cell function, techniques that allow the specific manipulation of local signalling provide an interesting opportunity to achieve a more targeted intervention. Displacement of specific isoforms of endogenous PDEs by the overexpression of their catalytically inactive mutant is one approach used in the laboratory. This strategy involves engineering specific PDE isoforms by introducing a single point mutation in their catalytic site. The overexpression of these constructs results in the displacement of the endogenous active PDE isoform from its original localisation within the cell. The technique was used to explore the role of specific anchored PDE isoforms in the compartmentalisation of CN signalling. Compared to the siRNA approach described above, the expression of catalytically inactive PDEs allows the role of PDE subcellular localisation in the regulation of a specific cellular function to be directly assessed. The first study to use this technique generated catalytically inactive PDE4A4, PDE4B1, PDE4C2, and PDE4D3 to identify the role of specific tethered PDE4 isoforms in regulating cAMP and PKA-dependent phosphorylation in resting COS1 cells. The study successfully showed that PDE4C2 and PDE4D3 isoforms are constitutively phosphorylated by PKA type-II, and as they are tethered in a complex including AKAP450 and PKA type-II, they play a central role in regulating the activation of AKAP450-bound PKA type-II at the perinuclear region [60]. In another study, catalytically inactive mutants of PDE2A, PDE3A2, and PDE4D3 were overexpressed in neonatal rat cardiac myocytes to investigate the role of different pools of cAMP in the regulation of cardiac myocyte hypertrophic growth. It was successfully shown that the displacement of PDE4D3 and PDE3A2 results in cardiac myocyte hypertrophy, whereas the displacement of PDE2A counteracts the hypertrophic growth induced by chronic β-adrenergic stimulation [47].

As previously discussed, different PDEs can localise to different subcellular sites—anchored to AKAPs or via interaction with other proteins. For example, PDE4D3, together with PKA, is localised by the mAKAP-organised signalosome in the perinuclear region [31], and via interaction with AKAP450, PDE4D3 localises at the centrosomes [61]. PDE4A4/5 interact with plasma membrane receptors [62], and PDE4D3 tethered together with PKA via interaction with AKAP18δ localises at aquaporin-2 vesicles, regulating vasopressin-mediated water reabsorption [63]. As mentioned above, PDE4D5 can form a complex with β-arrestin, and the PDE4D5-β-arrestin complex plays an important role in the activation of ERK signalling [45]. Therefore, another obvious strategy to achieve selective local manipulation of PDE activity involves the disruption of the interaction between the PDE and the protein interacting partner. In this approach, a short peptide or small molecule is used to target the interaction surface between the PDE and the anchoring protein. In a proof of principle experiment, this strategy was used to disrupt the interaction of PDE4D5 with HSP20, a heat shock protein with a role in protecting from cardiac dysfunction induced by sepsis and endotoxin in mice via PKA-dependent phosphorylation. In this study, selective local inhibition of PDE activity was achieved by using a synthetic peptide encompassing a short stretch of amino acid residues from the PDE4D5 sequence involved in binding to HSP20, and resulted in attenuated β-agonist-induced hypertrophy in neonatal cardiomyocytes [64,65]. As another example, PDE3B was found to interact with EPAC1 and p84, a phosphoinositide 3-kinase y (PI3Kγ) regulatory subunit. A cell permeable peptide was used to disrupt PDE3B–EPAC1 binding in human aortic endothelial cells (HAEC). The study demonstrates that manipulation of EPAC1–PDE3B interaction results in altered cAMP signalling, thus affecting EPAC1-mediated activation of PI3Kγ and dynamic cAMP-dependent regulation of HAEC cells adhesion, spreading, and tubule formation [66].

## 5. Conclusions

Cyclic nucleotide PDEs play an important role in cellular signalling by degrading cAMP and cGMP. Due to their diverse distribution in different tissues—at the cellular and subcellular levels—and their major role in regulating cellular function, PDEs have been the focus of intense research as potential therapeutic targets. The limitations with currently available pharmacological PDE inhibitors—mainly due to their lack of isoform selectivity—urge the development of new approaches to achieve effective and specific PDE inhibition for experimental work and clinical use. Emerging understanding of CN compartmentalised signalling, of the role of individual signalosomes, and the specific signalling and cellular responses regulated by individual PDE isoforms is providing the background knowledge and rationale for the development of new PDE targeting strategies, which in the future may deliver novel, more effective, and specific therapeutics.
